# Genomic alterations related to HPV infection status in a cohort of Chinese prostate cancer patients

**DOI:** 10.1186/s40001-023-01207-2

**Published:** 2023-07-17

**Authors:** Bin Lang, Chen Cao, Xiaoxiao Zhao, Yi Wang, Ying Cao, Xueying Zhou, Tong Zhao, Yuyan Wang, Ting Liu, Wenjia Liang, Zheng Hu, Xun Tian, Jingjing Zhang, Yongji Yan

**Affiliations:** 1 Peking University Health Science Center-Macao Polytechnic University Nursing Academy, Macao Polytechnic University, Macao, 999078 China; 2 Department of Obstetrics and Gynecology, Academician Expert Workstation, The Central Hospital of Wuhan, Tongji Medical College, Huazhong University of Science and Technology, Wuhan, 430014 Hubei China; 3 Department of Pathology, The Central Hospital of Wuhan, Tongji Medical College, Huazhong University of Science and Technology, Wuhan, 430014 Hubei China; 4 Operating Room, Zhongnan Hospital of Wuhan University, Wuhan, 430071 Hubei China; 5 Department of Obstetrics and Gynecology, The First Affiliated Hospital, Sun Yat-Sen University, Guangzhou, 510000 Guangdong China; 6 Department of Obstetrics and Gynecology, Women and Children’s Hospital Affiliated to Zhongnan Hospital of Wuhan University, Wuhan, 430071 Hubei China; 7 Department of Radiation and Medical Oncology, Zhongnan Hospital of Wuhan University, Wuhan, 430071 Hubei China; 8 Hubei Key Laboratory of Tumor Biological Behaviors, Zhongnan Hospital of Wuhan University, Wuhan, 430071 Hubei China; 9 Hubei Cancer Clinical Study Center, Zhongnan Hospital of Wuhan University, Wuhan, 430071 Hubei China; 10 Department of Gynecology and Oncology, Zhongnan Hospital of Wuhan University, Wuhan, 430062 Hubei China; 11 Department of Urology, Dongzhimen Hospital, Beijing University of Chinese Medicine, Beijing, 100700 China

**Keywords:** Capture sequencing, Human papillomavirus, Prostate cancer, Whole-exome sequencing

## Abstract

**Background:**

Human papillomavirus (HPV) has been proposed as a potential pathogenetic organism involved in prostate cancer (PCa), but the association between HPV infection and relevant genomic changes in PCa is poorly understood.

**Methods:**

To evaluate the relationship between HPV genotypes and genomic alterations in PCa, HPV capture sequencing of DNA isolated from 59 Han Chinese PCa patients was performed using an Illumina HiSeq2500. Additionally, whole-exome sequencing of DNA from these 59 PCa tissue samples and matched normal tissues was carried out using the BGI DNBSEQ platform. HPV infection status and genotyping were determined, and the genetic disparities between HPV-positive and HPV-negative PCa were evaluated.

**Results:**

The presence of the high-risk HPV genome was identified in 16.9% of our cohort, and HPV16 was the most frequent genotype detected. The overall mutational burden in HPV-positive and HPV-negative PCa was similar, with an average of 2.68/Mb versus 2.58/Mb, respectively, in the targeted whole-exome region. HPV-negative tumors showed a mutational spectrum concordant with published PCa analyses with enrichment for mutations in *SPOP*, *FOXA1*, and *MED12*. HPV-positive tumors showed more mutations in *KMT2C*, *KMT2D* and *ERCC2*. Copy number alterations per sample were comparable between the two groups. However, the significantly amplified or deleted regions of the two groups only partially overlapped. We identified amplifications in oncogenes, including *FCGR2B* and *CCND1*, and deletions of tumor suppressors, such as *CCNC* and *RB1*, only in HPV-negative tumors. HPV-positive tumors showed unique deletions of tumor suppressors such as *NTRK1* and *JAK1*.

**Conclusions:**

The genomic mutational landscape of PCa differs based on HPV infection status. This work adds evidence for the direct involvement of HPV in PCa etiology. Different genomic features render HPV-positive PCa a unique subpopulation that might benefit from virus-targeted therapy.

**Supplementary Information:**

The online version contains supplementary material available at 10.1186/s40001-023-01207-2.

## Background

Prostate cancer (PCa) is the second most common cancer in men worldwide [1]. The incidence and mortality have increased rapidly in China during the last decade [2]. The firmly established risk factors include advanced age, family history of this malignancy, certain genetic mutations (e.g., *BRCA1* and *BRCA2*) and conditions (e.g., Lynch syndrome). High-risk human papillomavirus (hrHPV) infection has also been suggested as a risk factor for PCa [3]. To date, studies have identified the presence of hrHPV in PCa tissue and an increased odds ratio (OR) of HPV infection in PCa compared with controls [4]. However, the dominant detection method of previous studies was PCR-based, focusing only on specific HPV types rather than comprehensive detection of all hrHPV types, which may result in variations in reported HPV infection rates across studies [5].

Further investigations revealed that HPV infection can impact the development of PCa by triggering chronic inflammatory processes, resulting in DNA damage [6, 7]. HPV-driven cancers possess a widely distributed exogenous virus that creates a specific mutagenic environment. HPV genes can disrupt crucial pathways responsible for preserving genome integrity. Eventually, this disruption gives rise to additional genetic mutations, allowing the initiation of cancer [8]. PCa is regarded as being closely associated with accumulation of somatic mutations in the prostate epithelial cell genome, and the Cancer Genome Atlas (TCGA) taxonomy [9] for PCa is based on seven important oncogenic drivers, including *ERG*, *ETV1*, *ETV4*, *FLI1*, *SPOP*, *FOXA1*, and *IDH1*. In total, 68.4% of Chinese PCa cases can be attributed to one of the TCGA taxonomies [10]. However, whether HPV infection in PCa causes genomic alterations and subsequently contributes to tumor initiation and progression has not been fully investigated.

In this study, we investigated hrHPV prevalence using capture sequencing in 59 Chinese PCa patients, and performed whole-exome sequencing (WES) to evaluate the association between the HPV status and genomic alterations. This study aimed to characterize the distinct molecular landscape of hrHPV-positive and hrHPV-negative PCas. We believe that elucidating the causative role of hrHPV in PCa will help to strengthen calls for cancer screening and vaccination programs.

## Methods

### Patients and samples

The study was approved by the Ethics Committee of the Central Hospital of Wuhan in China (WHZXKYL2022-047). We recruited 59 PCa patients admitted to our hospital from January 2019 to December 2020. Formalin-fixed, paraffin-embedded (FFPE) tissue blocks were examined by an experienced pathologist to select tumor samples with malignant cell purities over 70% and adjacent normal tissues. All patients provided signed informed consent before enrollment.

For each FFPE sample, DNA was extracted using E.A.N., an FFPE DNA Kit (Omega Biotek) according to the manufacturer’s protocol. The DNA concentration was quantified using a Qubit 4.0 Fluorometer. The fragment length and degradation were assessed by a Qsep100 bioanalyzer (BIOptic). The DNA was stored at – 20 °C before use.

### HPV genotyping by capture sequencing

Capture sequencing was performed as described previously [11]. Briefly, we designed a custom panel containing the whole-genome sequences of 15 types of hrHPV, and ordered a biotinylated RNA probe library from IDT (IDT, USA).

DNA (250 ng) from tumor samples was sheared to 250–350 bp by a Bioruptor Pico (Diagenode). After purification using Agencourt AMPure XP beads (Beckman), whole-genome libraries were constructed using a TargetSeq Enrichment Kit (iGeneTech). Hybridization was performed at 65 °C for 16 h. After capture and PCR amplification, the HPV libraries were analyzed using Qubit4.0 and Qsep100 bioanalyzers, and sequenced using the Illumina HiSeq2500 platform.

Data were submitted to our in-house pipeline VIPA [12] for HPV genotype and integration detection. This included the following: (i) quality control; (ii) reference preparation. A human reference genome (GRCh38.p12) was downloaded from UCSC (http://genome.ucsc.edu/), and HPV genome references was downloaded from the PaVE database (http://pave.niaid.nih.gov); (iii) alignment to the mixed human and 15 types of hrHPV references with BWA-MEM [13] to detect virus genomes; (iv) remapping of the clean reads to mixed human–virus references to identify breakpoints; (v) junction positions were annotated by ANNOVAR (V2017-07-17) [14] as integration breakpoints.

### WES sample processing

Two micrograms of DNA from all tumor and adjacent normal samples was sent to BGI company (Wuhan, China) for library preparation and sequencing. The Agilent SureSelect Human All Exon V6 kit (Agilent Technologies, 60.33 Mb target region) was used for WES capture experiments according to the manufacturer’s recommendations. Specifically, a Bioruptor Pico shearing system was used for fragmentation to generate 200–300 bp fragments. Next, liquid-phase hybridization was performed to selectively enrich DNA fragments using biotin-labeled probes. After library quantification, PE100 data were generated using a BGI DNBSEQ platform.

### WES alignment and variant calling

Trimmed paired-end reads were aligned to the UCSC hg38 reference genome using BWA-MEM. Picard tools and SAMtools [15] were employed to remove PCR duplicate reads and deal with alignment files. Bam files were locally realigned using Genome Analysis Toolkit (GATK) to improve accuracy [16]. Somatic mutations in tumor–control paired samples were detected by GATK Mutect2 [17] and annotated by ANNOVAR. The Mutation Annotation Format (MAF) of somatic variants was visualized by using the maftools [18] R package.

### Significant CNV detection

Somatic copy number variation (CNV) was called with FACETS [19] using deduplicated mapping bam files for each paired sample. FACETS provides estimates of copy number based on comparing binned read depths to the reference genome. The CNVs were annotated using GISTIC2.0 [20] to generate focal-level CNVs for the cohort with G-Score and FDR *Q* values used to indicate the significance of the CNVs identified.

### Statistical analysis

Statistical analyses were performed using Prism 9 (GraphPad) and SPSS Statistics v.26 (IBM). One-way ANOVA was used to assess the relationship between mean age and clinical characteristics. Molecular alterations were compared using Chi-squared Fisher’s exact test for categorical variables and Welch’s *t* test for continuous variables. *P* values < 0.05 were considered statistically significant.

## Results

### Patient characteristics

Of the 59 PCa patients included in this study, the mean age was 72.83 $\pm$ 6.22 years. The baseline characteristics are shown in Table 1. The mean age of the patients showed no significant relationship with the tPSA value, fPSA/tPSA ratio, ISUP group, T stage, N stage, or M stage. There was no significant difference in the distribution of all clinical features between the higher age group and the lower age group, except for a higher fPSA/tPSA ratio detected in the age group over 70 years (*P* = 0.010).

**Table 1:** Clinical characteristics of cohort

Characteristics	Mean age, yr, $\pm$ SD	Age group, yr, no. (%)		Total, no
		< 70	≥ 70	
	72.83 ± 6.22	15 (25.4)	44 (74.6)	59
tPSA (ng/ml)				
< 4	73 ± 2.71	0 (0.0)	4 (100.0)	4
4–10	73.33 ± 6.92	3 (25.0)	9 (75.0)	12
> 10	72.65 ± 6.44	11 (31.4)	24 (68.6)	35
NA	/	1 (12.5)	7 (87.5)	8
	*P* = 0.953	*P* = 0.610		
fPSA/tPSA				
< 0.16	71.92 ± 6.05	13 (36.1)	23 (63.9)	36
≥ 0.16	75.71 ± 6.12	0 (0.0)	14 (100.0)	14
NA	/	1 (11.1)	8 (88.9)	9
	*P* = 0.060	*P* = 0.010		
ISUP group				
1	74.64 ± 8.52	2 (18.2)	9 (81.8)	11
2	72.64 ± 6.34	5 (35.7)	9 (64.3)	14
3	73.15 ± 3.67	2 (15.4)	11 (84.6)	13
4	70.7 ± 6.88	4 (40.0)	6 (60.0)	10
5	72.82 ± 5.67	2 (18.2)	9 (81.8)	11
	*P* = 0.721	*P* = 0.571		
T stage				
1	75.6 ± 11.19	2 (40.0)	3 (60.0)	5
2	73.32 ± 5.37	6 (21.4)	22 (78.6)	28
3	70.38 ± 5.25	4 (25.0)	12 (75.0)	16
4	74 ± 6.62	3 (30.0)	7 (70.0)	10
	*P* = 0.265	*P* = 0.809		
N stage				
0	72.65 ± 6.15	13 (25.5)	38 (74.5)	51
1	74.80 ± 7.69	1 (20.0)	4 (80.0)	5
X	72.67 ± 6.81	1 (33.3)	2 (66.7)	3
	*P* = 0.767	*P* = 1.000		
M stage				
0	72.76 ± 6.247	15 (25.9)	43 (74.1)	58
1	77 ± 0.0	0 (0.0)	1 (100.0)	1
	*P* = 0.504	*P* = 1.000		

In the tPSA, ISUP group, T stage, and N stage group, *P* values for mean age were determined using one-way ANOVA analysis, and *P* values for age groups were determined using Fisher’s exact test

In the fPSA/tPSA, M stage group, *P* values for mean age were determined using 2-tailed unpaired *t-*test, *P* values for age groups were determined using Fisher’s exact test

### HPV genotypes and clinical characteristics relative to HPV status

HPV genotyping results showed that 10 patients (16.9%) were HPV positive. HPV16 was the most frequent genotype detected in eight cases (13.6%). We found the presence of both single-type infection and coinfection, including single HPV16 (11.9%), double HPV16/98 (1.7%), single HPV18 (1.7%) and multiple HPV26/51/66 (1.7%) infections (Additional file 1: Figure S1A).

The median age of the patients showed no statistically significant difference between the HPV-positive and HPV-negative groups (Additional file 1: Figure S1B). As shown in Table 2, the distribution of the T stage between the two groups did differ. The highest prevalence of HPV was in the T1 stage (3/5, 60%), followed by the T4 stage (3/10, 30%), but no trend was observed for higher T stage corresponding to higher HPV infection rates. Additionally, the frequency distributions of age, ISUP group, N stage, and M stage between the positive and negative groups were not significant.

**Table 2:** Distribution of HPV infection status by age, ISUP group, and stage in this cohort

Characteristics	HPV-positive No. (%)	HPV-negative No. (%)	Total No
	10 (16.9)	49 (83.1)	59
Age (yr)			
< 70	4 (26.7)	11 (73.3)	15
≥ 70	6 (13.6)	38 (86.4)	44
	*P* = 0.257		
ISUP group			
1	4 (36.4)	7 (63.6)	11
2	2 (14.3)	12 (85.7)	14
3	0 (0.0)	13 (100.0)	13
4	2 (20.0)	8 (80.0)	10
5	2 (18.2)	9 (81.8)	11
	*P* = 0.188		
T stage			
1	3 (60.0)	2 (40.0)	5
2	3 (10.7)	25 (89.3)	28
3	1 (6.3)	15 (93.8)	16
4	3 (30.0)	7 (70.0)	10
	*P* = 0.024		
N stage			
0	7 (13.7)	44 (86.3)	51
1	2 (40.0)	3 (60.0)	5
X	1 (33.3)	2 (66.7)	3
	*P* = 0.156		
M stage			
0	10 (17.2)	48 (82.8)	58
1	0 (0.0)	1 (100.0)	1
	*P* = 1.000		

In the ISUP group, T stage, and N stage group, *P* values for mean age were determined using one-way ANOVA analysis, and *P* values for HPV infection status were determined using Fisher’s exact test

In the age and M stage group, *P* values for mean age were determined using Welch’s *t*-test, and *P* values for HPV infection status were determined using Fisher’s exact test

We performed pipeline VIPA and found no HPV DNA integration event in our data set.

### Detection of somatic aberrations

WES was performed on 59 tumor–adjacent normal pairs. All samples had at least 60.12 Mb of target exome region covered with a median depth of 145.54$\times$ (range: 18.31–268.47$\times$) for tumor samples and 107.17$\times$ (range: 32.76–142.03$\times$) for adjacent normal samples (Additional file 4: Table S1). Collectively, the 59 samples contained 24,058 somatic mutations, including 4902 missense, 428 nonsense, 2254 silent, 315 splice-site, four nonstop mutations, 559 frameshift insertions and deletions (indels) and 406 in-frame indels. Eight hypermutant samples showed outlier mutation frequencies (>600 mutants per sample). The average mutation density was 6.76 mutations per Mb across all tumors, and 2.59 when the hypermutant tumors were excluded (Additional file 5: Table S2). At all targeted bases, we did not detect a significant difference in overall mutation rates by HPV status (HPV-positive 2.68/Mb, HPV-negative 2.58/Mb, *P* = 0.891) (Additional file 2: Figure S2).

**Fig. 1:**
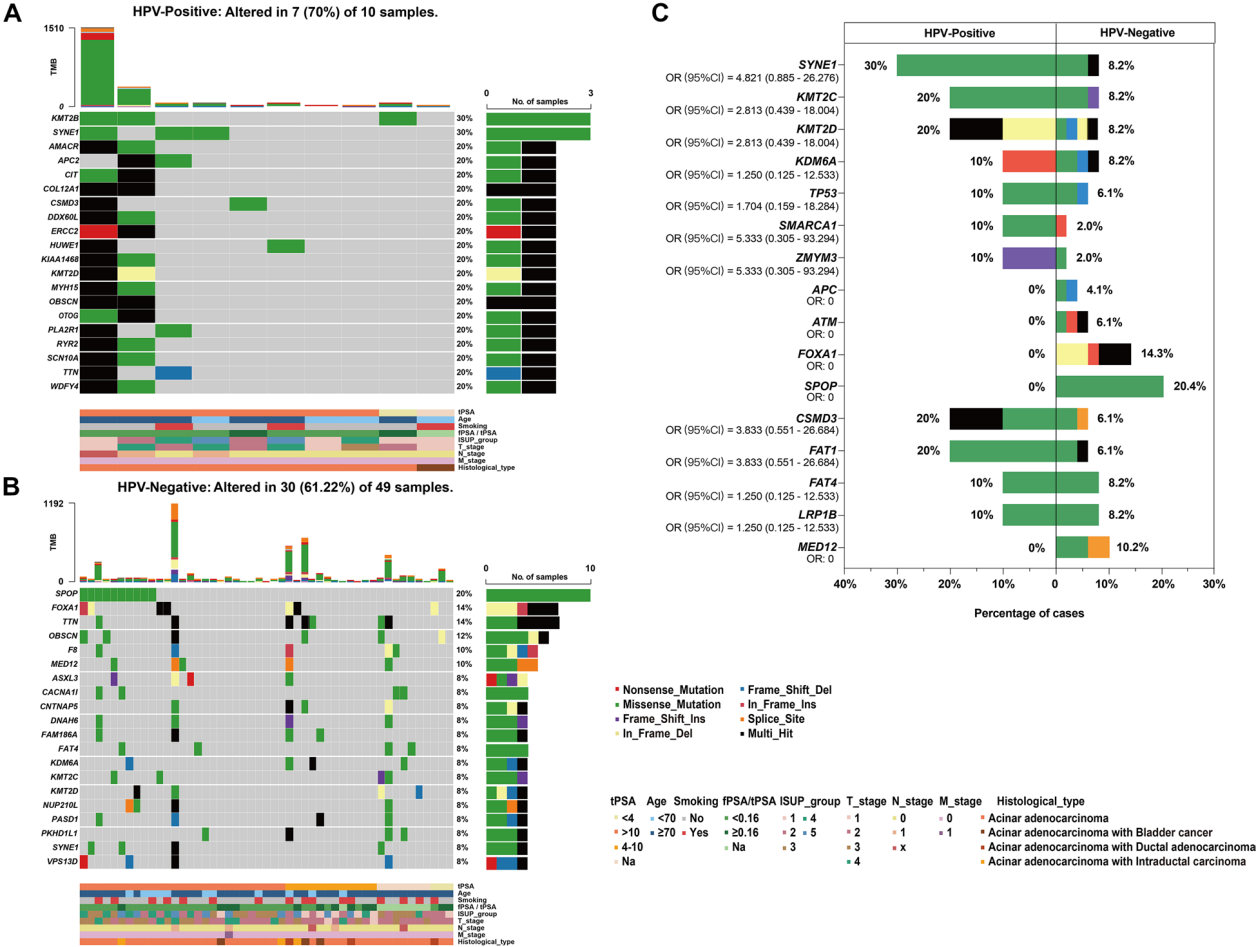
The landscape of somatic mutation profiles in PCa samples. Mutation information of each gene in each HPV-positive PCa sample (**A**) and HPV-negative PCa sample (**B**) is shown in the waterfall plot. Each column represents a tumor with the bar graph at the top depicting the number of alterations per sample. Each Oncoprint row shows alterations for each gene. The bar graph on the right of the panel shows the number and distribution of alterations per gene. Different colors with specific annotations at the bottom depict the various mutation types and clinical features. **C** A stacked bar plot shows the differences in the SMGs mutation of HPV-positive PCa versus HPV-negative PCa. OR: odds ratio for HPV (Positive/Negative)

Overall, the most common genomic alterations in PCa were mutations in *SPOP* (17%), followed by *TTN* (15%), *OBSCN* (14%), *FOXA1* (12%), and *SYNE1* (12%). The findings are summarized in Additional file 3: Figure S3. As illustrated in the waterfall map in Fig. 1A, the somatic mutation was altered in seven of ten HPV-positive PCa patients (70%). *KMT2B* and *SYNE1* were the most frequently mutated genes. Figure 1B shows that the somatic mutation was altered in 30 of 49 HPV-negative PCa patients (61.22%). *SPOP*, *FOXA1*, *TTN*, and *OBSCN* were the four most frequently mutated genes, with the frequencies of 20.4%, 14.3%, 14.3%, and 12.2%, respectively. Only four of the top 20 mutated genes were shared by the two groups.

Next, the mutation differences of genes were compared between these two groups. Using Cancer Gene Census (CGC), a curated list of 736 known cancer genes [21], we compared cancer genes in the most frequently mutated gene list (> 8% of samples) in our cohort. Genes that have been previously reported as significantly mutated genes (SMGs) of Asian PCa [10] were also compared. Among the 11 SMGs, HPV-positive patients showed higher alteration frequencies in *SYNE1*, *KMT2C*, and *KMT2D*. *SPOP*, *FOXA1*, *ATM*, and *APC* mutations were observed only in the HPV-negative group. Among the other five CGC genes, HPV-positive patients showed a higher alteration frequency for *CSMD3* and *FAT1*, and *MED12* mutation was observed only in the HPV-negative group, but these differences did not reach statistical significance (*P* $>$ 0.05) (Fig. 1C).

### CNV analysis

**Fig. 2:**
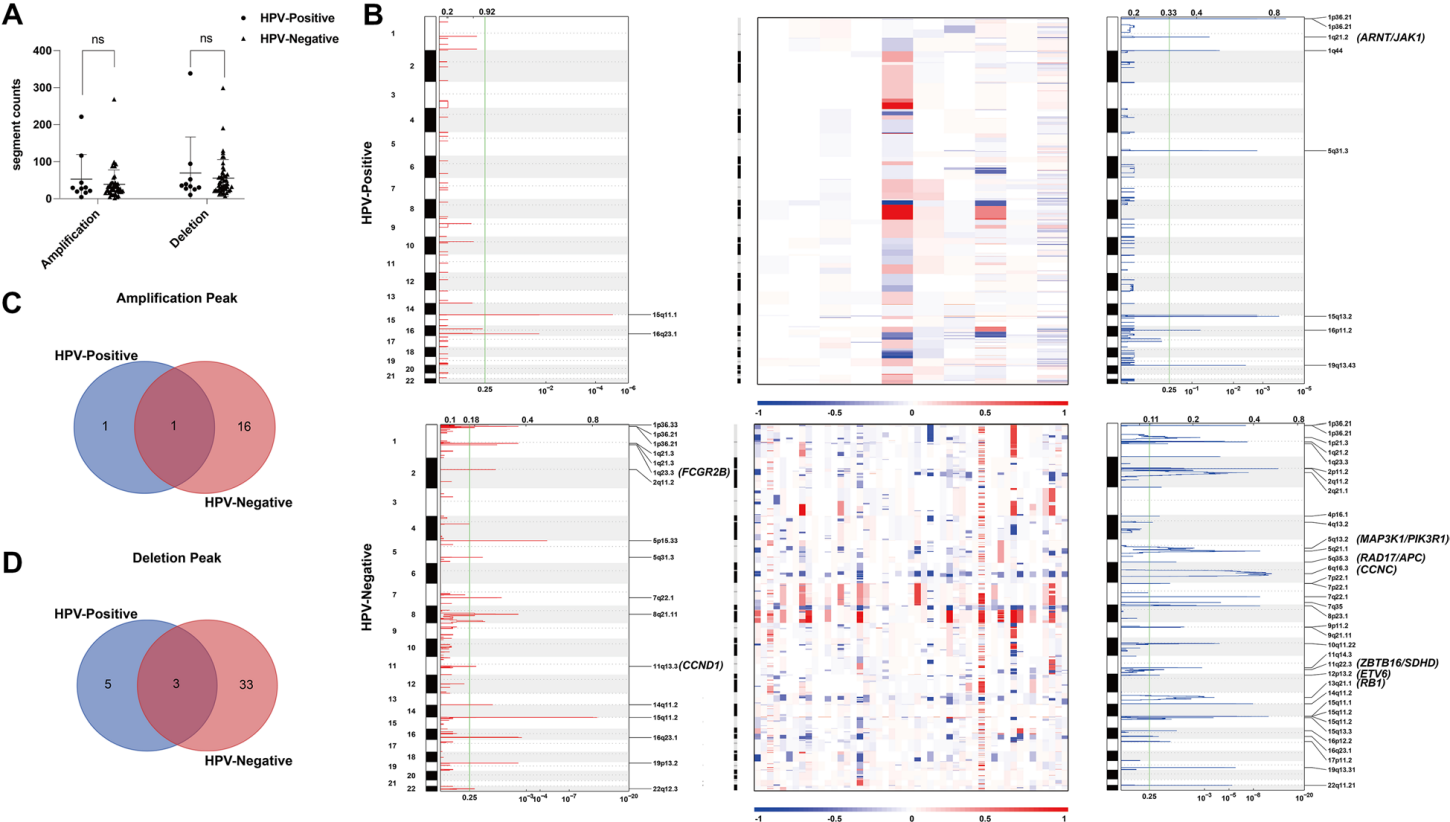
Distribution of CNVs in our cohort. **A** Comparison of CNVs between the HPV-positive group and HPV-negative group. *P* = 0.840 is determined by the Mann–Whitney *U* test in copy number amplifications and *P* = 0.824 in copy number deletions. **B** Focal-level somatic CNV events. Red and blue denoted amplification and deletion, respectively. Chromosomal locations of peaks of significantly recurring focal amplification and deletion are filtered by FDRs. Peaks are annotated with amplification of candidate oncogenes or deletion of TSGs by cytoband (1q23.3(*FCGR2B*), 11q13.3(*CCND1*), 1q21.2(*ARNT/JAK1*), 5q13.2(*MAP3K1/PIK3R1*), 5q35.3(*RAD17/APC*), 6q16.3(*CCNC*), 11q22.3(*ZBTB16/SDHD*), 12p13.2(*ETV6*), and 13q21.1(*RB1*)). Heatmap of the CNAs of 10 HPV-positive samples (top) and 49 HPV-negative samples (bottom) in units of log2 (tumor/adjacent non-tumor) along the chromosomes. **C**, **D** Venn diagrams showing comparisons of recurrent peaks between the HPV-positive group and HPV-negative group

A total of 5719 CNVs were identified in the 59 PCa samples, including 2358 amplifications (526 in the positive group and 1832 in the negative group) and 3361 deletions (683 in the positive group and 2678 in the negative group). The CNVs per sample are depicted and compared in Fig. 2A. No differences were observed in either amplifications (*P* = 0.840) or in deletions (*P* = 0.824) between these two groups. The most frequent gains were in chromosomes 7p, 7q, and 8q and losses in chromosomes 8p and 16q, similar in the two groups (Fig. 2B). In total, we identified two amplification peaks and eight deletion peaks in the HPV-positive group, and 17 amplification peaks and 36 deletion peaks in the HPV-negative group (Fig. 2B and Additional file 6: Table S3). When comparing these recurrent peaks, we found one common amplification peak and three common deletion peaks between the two different HPV status groups (Fig. 2C, D). We found a clear difference in the CGC genes contained in each wide peak (Fig. 2B and Additional file 6: Table S3). No amplification of oncogenes was identified in the HPV-positive group, but two amplifications of oncogenes were identified in the HPV-negative group: *FCGR2B* (1q23.3, five cases) and *CCND1* (11q13.3, five cases). Twenty-nine deletions of key tumor suppressor genes (TSGs) were identified in the HPV-positive group, including *ARNT* (1q21.2, two cases), *JAK1* (1q21.2, two cases), and *NOTCH2* (1q21.2, two cases); 22 deletions of TSGs were identified in the HPV-negative group, including *CCNC* (6q16.3, 22 cases), *RB1* (13q21.1, 17 cases), *RAD17* (5q35.3, 11 cases), *MAP3K1* (5q13.2, 11 cases), and *PIK3R1* (5q13.2, 11 cases). Interestingly, in one of the common deletion peaks 1q21.2, all 29 TSGs, including *ARNT*, *JAK1*, and *NOTCH2*, were only contained in the HPV-positive group peak (not in the HPV-negative group peak).

**Fig. 3:**
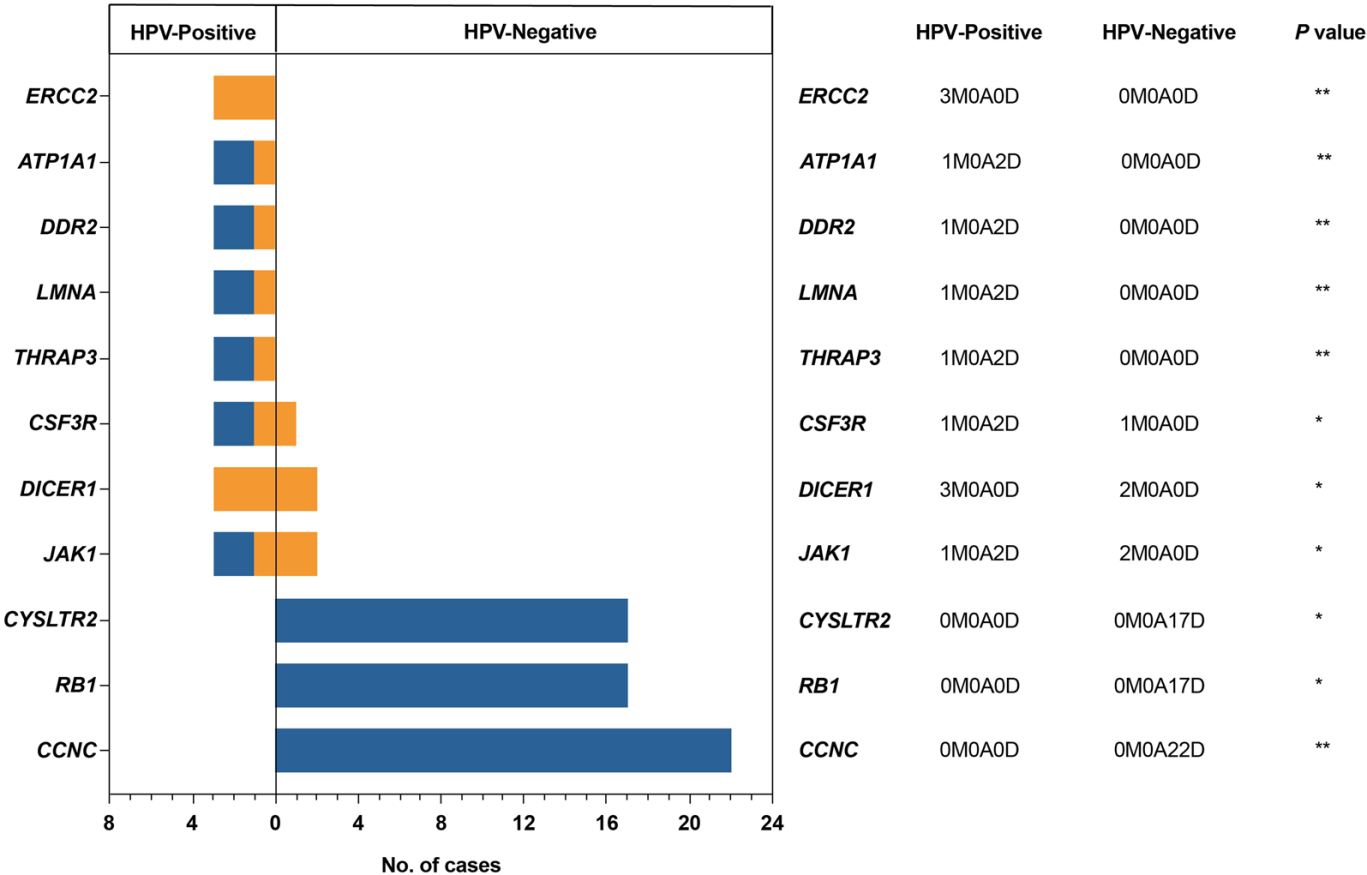
Cohort comparison analysis. Differentially mutated genes between HPV-positive group and HPV-negative group are displayed as a bar plot. Orange and blue denote mutation and deletion, respectively. The adjacent table includes the number of samples in each group with the alterations in the highlighted gene. M, nonsynonymous mutation; A, amplification; D, deletion. *P* value indicates the significance threshold. *P < 0.05, **P < 0.01 was determined by Fisher’s exact test

We then combined mutations and focal CNVs to define an enlarged list of putative HPV-related genes. We implemented the mafCompare function to identify differentially mutated CGC genes between the HPV-positive and HPV-negative groups, with the mutation load for each gene being compared by Fisher’s exact tests. Comparisons of these two groups revealed 55 differentially altered genes (*P* $<$ 0.05) (Additional file 7: Table S4). By utilizing PRISM dataset (https://depmap.org/portal/prism/) [22], we attempted to identify potential drug targets among the 55 genes mentioned above. In the HPV-positive group, 13 targetable mutations and copy number aberrations were found, while in the HPV-positive group, one targetable copy number deletion was identified. These genes have been individually annotated in Additional file 7: Table S4. Figure 3 shows the comparison of 11 genes with ≥ three alterations (nonsynonymous mutation, copy number amplification or copy number deletion) among the 59 PCa samples. Among them, *ERCC2*, *ATP1A1*, *DDR2*, *LMNA*, and *THRAP3* were significantly enriched only in the HPV-positive group. *CCNC*, *RB1*, and *CYSLTR2* were significantly enriched only in the HPV-negative group.

## Discussion

HPV infection is the most common sexually transmitted infection worldwide. Although the vast majority of infections disappear within 1 to 2 years, persistent hrHPV infection is responsible for virtually all cervical and anal cancers, 70% of vaginal and vulvar cancers, and a large percentage of penile cancers [23].

The majority of previous studies on assessment of HPV prevalence in PCa applied PCR methods that can only detect a limited number of HPV genotypes. Additional factors such as the multifocal feature of PCa and detection using biopsy specimens may contribute to poor specificity for HPV detection. Several studies examined hrHPV infection in PCa, and the frequency varied in a vast range from 2 to 75% [24]. In this study, we applied a hybrid capture-based NGS assay to FFPE samples, which enlarged the pool of HPV types to search for 15 types of hrHPV and effectively enabled us to determine HPV genotypes and integration status with high sensitivity. Moreover, capture sequencing can robustly detect multiple hrHPV infections [25]. In our study, HPV DNA was detected in 16.9% of patients with PCa, with HPV16 being the most frequent genotype and 20% of HPV-positive cases were multiple-type coinfections. These data indicate that the burden of HPV infection in men and the male genitourinary tract might serve as a reservoir of HPV transmission to women.

Many studies have focused on the Western population, whereas only a few have profiled molecular signatures in PCas in Asian. We performed WES to analyze genomic alterations in Chinese PCa patients and conducted comparative analysis of genomic differences between HPV-positive and HPV-negative groups. The mutational spectrum in HPV-negative PCa with enrichment for mutations in *SPOP*, *FOXA1*, and *MED12* is very similar to that previously reported for PCa [26]. Of note, no *SPOP*, *FOXA1*, or *MED12* mutations were observed in the HPV-positive PCa group. Preclinical studies have revealed that mutations in *SPOP* promote genetic instability in PCa and drive prostate tumorigenesis through coordinated regulation of PI3K/mTOR and AR signaling [27]. *FOXA1* is essential for prostate organogenesis and functions as an oncoprotein that increases transcription of androgen receptor (AR) to drive metastatic progression [28]. Recent evidence suggests that patients with *FOXA1* mutations have less favorable prognosis [10]. It has also been proposed that *MED12* mutations in PCa may disrupt the androgen signaling pathway and CDK8-dependent transcriptional regulation of p53 [26].

In contrast, HPV-positive PCa showed distinct alterations with top mutations in *KMT2B*, *CSMD3*, *ERCC2*, and *KMT2D*. A recent study reported that *KMT2B* facilitates cervical cancer metastasis and angiogenesis by upregulating EGF expression*. KMT2B*, along with *KMT2C* and *KMT2D*, belongs to the lysine methyltransferase 2 (KMT2) family. The KMT2 family members are important regulators of gene transcription in cancer [29]. Given the increasing focus on abnormal epigenetic regulation in cancer, further investigation of involvement of the KMT2 family in HPV-positive PCa is needed. ERCC group genes are key factors in DNA transcription and the nucleotide excision repair pathway, which is an important DNA repair mechanism. It has been demonstrated that deficiency in the DNA repair gene *ERCC2* has a central role in modulation of PCa susceptibility. *ERCC2* polymorphism was observed to be related to increased risk in the Asian PCa population [30].

Next, we found comparable average CNVs between the HPV-positive and HPV-negative groups. The number of recurrent amplification or deletion peaks was much lower in the HPV-positive group, and each wide peak contained a completely different composition of candidate cancer genes. Among the 29 deletions of key TSGs identified in the HPV-positive group, *JAK1* was previously found to promote sensitivity to docetaxel in PCa cells. Further drug–gene interaction analyses identified that combination therapy with *JAK1* inhibitors and docetaxel may be useful in PCa treatment. Loss-of-function *JAK1* mutations occur at high frequency in cancers with MSI and represent a potential pancancer adaptation of immune evasion [31]. *NTRK1*, another TSG deletion identified in the HPV-positive group, is recognized as a prognostic marker of primary PCa. Downregulation of *NTRK1* is linked to poor prognosis in PCa, and *NTRK1* expression correlates significantly with immune cell infiltration levels [32].

Our study presents novel somatic alterations in cancer-related genes in HPV-positive PCa, e.g., *ATP1A1*, *DDR2*, *LMNA*, *THRAP3*, *CSF3R*, and *DICER1*. To our knowledge, these mutations have not been previously described in PCa, and *DICER1* mutation is of particular interest. DICER1 syndrome [33] is an autosomal dominant familial tumor predisposition disorder involving a heterozygous *DICER1* germline mutation. Mutation of *DICER1* results in the susceptibility to a variety of malignant tumors.

Precision oncology in PCa is rapidly evolving. In this study, we focused on the specific genetic events in HPV-positive and HPV-negative PCa likely contribute to the distinct biologic behavior. Today, the HPV vaccination program has been extended to prevent increasing incidence of HPV-related cancers, and HPV-positive PCa as a unique subpopulation might benefit from HPV vaccination. Besides, genomic testing is increasingly common, as biomarker-guided therapies are approved for specific individuals harboring genetic alterations in DNA repair genes such as BRCA [34, 35]. Here, we identified a series of potential therapeutic targets through tissue-based sequencing. HPV-positive PCas were characterized by mutations in *KMT2C*, *KMT2D* and *ERCC2*. Copy number deletions in *NTRK1* and *JAK1* were also discovered. These driver genes may serve as therapeutic targets for HPV-positive PCa. Mutations in *SPOP*, *FOXA1*, and *MED12* were identified exclusively as driver genes in HPV-negative PCa as potential therapeutic targets. Additionally, these distinct genomic alterations identified in different HPV status subgroups, either individually or in combination as a panel, may serve as characteristic biomarkers for future liquid biopsies of HPV-positive or HPV-negative PCa [36]. These biomarkers could be utilized for early detection, treatment stratification, or recurrence monitoring. However, the therapeutic implications of these distinct gene aberrations in PCa require investigation and validation in future studies.

## Limitations

One primary limitation of this study was the small sample size of 59 patients in our cohort. Second, this is a retrospective study and the proportion of HPV-positive patients is relatively low. Therefore, expanding the sample size and conducting a prospective randomized trial are required to provide stronger evidence.

## Conclusion

Here, we provide HPV capture sequencing as a valuable approach for HPV-related PCa screening. The HPV-positive group had several genomic features that differed from the negative group in specific mutations and TSGs with CNV. These characteristics render HPV-positive PCa a unique subpopulation that might benefit from HPV vaccination and virus-targeted therapy. Furthermore, detecting HPV infection and mutation characteristics in PCa patients can guide personalized treatment, future studies of clinical implications of the observed mutations will be vital.

### Supplementary Information

**Additional file 1: Figure S1.** The HPV infection status of cohort. (A) The distribution of HPV single and coinfections in PCa. (B) The correlation between age and HPV infection status. *P* = 0.893 was determined by Welch’s *t*-test.

**Additional file 2: Figure S2.** The average mutation density between HPV-positive and HPV-negative PCa groups when the 8 hypermutant tumors were excluded. *P* = 0.891 was determined by Welch’s *t*-test.

**Additional file 3: Figure S3.** Oncoplot depicting the most recurrent somatic mutations in PCa cohort.

**Additional file 4: Table S1.** The summary of WES quality control.

**Additional file 5: Table S2.** The summary of somatic SNV and InDel.

**Additional file 6: Table S3.** CNV information in our cohort. Related to Fig. 3.

**Additional file 7: Table S4.** The summary of differentially altered genes between HPV-positvie and HPV-negative groups.

## Data Availability

All data generated or analyzed during the current study are available in the main text or the additional files. Sequencing data generated in this study are available through corresponding author Yan upon request.

## Abbreviations

PCa: Prostate cancer
TCGA: The Cancer Genome Atlas
HPV: Human papillomavirus
hrHPV: High-risk HPV
OR: Odds ratio
WES: Whole-exome sequencing
FFPE: Formalin-fixed, paraffin-embedded
CNV: Copy number variation
indels: Insertions and deletions
SMGs: Significantly mutated genes
TSG: Tumor suppressor genes
AR: Androgen receptor
PARP: Poly-ADP ribose polymerase
