# Correction: Genomic alterations related to HPV infection status in a cohort of Chinese prostate cancer patients

**DOI:** 10.1186/s40001-024-01935-z

**Published:** 2024-06-26

**Authors:** Bin Lang, Chen Cao, Xiaoxiao Zhao, Yi Wang, Ying Cao, Xueying Zhou, Tong Zhao, Yuyan Wang, Ting Liu, Wenjia Liang, Zheng Hu, Xun Tian, Jingjing Zhang, Yongji Yan

**Affiliations:** 1 Peking University Health Science Center-Macao Polytechnic University Nursing Academy, Macao Polytechnic University, Macao, 999078 China; 2 Department of Obstetrics and Gynecology, Academician Expert Workstation, The Central Hospital of Wuhan, Tongji Medical College, Huazhong University of Science and Technology, Wuhan, 430014 Hubei China; 3 Department of Pathology, The Central Hospital of Wuhan, Tongji Medical College, Huazhong University of Science and Technology, Wuhan, 430014 Hubei China; 4 Operating Room, Zhongnan Hospital of Wuhan University, Wuhan, 430071 Hubei China; 5 Department of Obstetrics and Gynecology, The First Affiliated Hospital, Sun Yat-Sen University, Guangzhou, 510000 Guangdong China; 6 Department of Obstetrics and Gynecology, Women and Children’s Hospital Affiliated to Zhongnan Hospital of Wuhan University, Wuhan, 430071 Hubei China; 7 Department of Radiation and Medical Oncology, Zhongnan Hospital of Wuhan University, Wuhan, 430071 Hubei China; 8 Hubei Key Laboratory of Tumor Biological Behaviors, Zhongnan Hospital of Wuhan University, Wuhan, 430071 Hubei China; 9 Hubei Cancer Clinical Study Center, Zhongnan Hospital of Wuhan University, Wuhan, 430071 Hubei China; 10 Department of Gynecology and Oncology, Zhongnan Hospital of Wuhan University, Wuhan, 430062 Hubei China; 11 Department of Urology, Dongzhimen Hospital, Beijing University of Chinese Medicine, Beijing, 100700 China

**Correction: European Journal of Medical Research: 2023 (28) 239** 10.1186/s40001-023-01207-2

The grant number “Polytechnic University (RP/ESCSD-05/2021)” has been included in this correction, and the original article [1] has been corrected.

## References

[1] Lang B, Cao C, Zhao X, Wang Y, Cao Y, Zhou X, Zhao T, Wang Y, Liu T, Liang W, Hu Z, Tian X, Zhang J, Yan Y (2023). Genomic alterations related to HPV infection status in a cohort of Chinese prostate cancer patients. Eur J Med Res.

