# Supplementary material for: Genomic alterations related to HPV infection status in a cohort of Chinese prostate cancer patients
Source: Eur J Med Res. 2023 Jul 17;28:239. doi: 10.1186/s40001-023-01207-2 (PMC10351112; doi:10.1186/s40001-023-01207-2)
Supplement: Supplementary file 1 — Additional file 1: Figure S1. The HPV infection status of cohort. (A) The distribution of HPV single and coinfections in PCa. (B) The correlation between age and HPV infection status. P = 0.893 was determined by Welch’s t-test. [file 40001_2023_1207_MOESM1_ESM.docx]

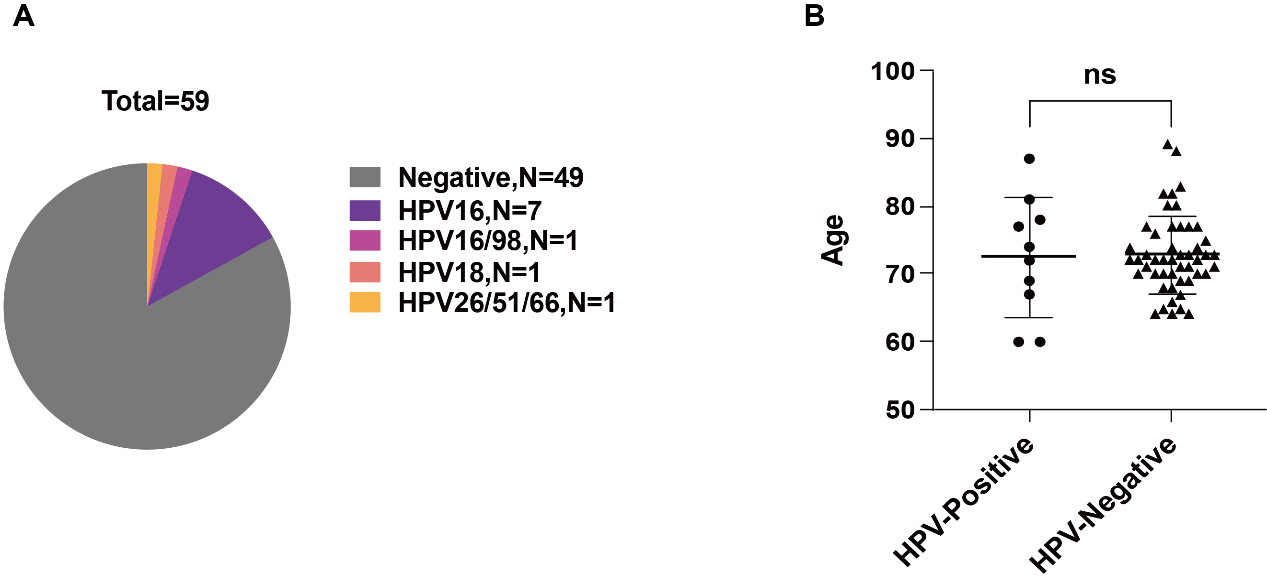


Figure S1. The HPV infection status of cohort. (A) The distribution of HPV single and coinfections in PCa. (B) The correlation between age and HPV infection status. *P* = 0.893 was determined by Welch’s t-test.
