# Supplementary material for: Genomic alterations related to HPV infection status in a cohort of Chinese prostate cancer patients
Source: Eur J Med Res. 2023 Jul 17;28:239. doi: 10.1186/s40001-023-01207-2 (PMC10351112; doi:10.1186/s40001-023-01207-2)
Supplement: Supplementary file 2 — Additional file 2: Figure S2. The average mutation density between HPV-positive and HPV-negative PCa groups when the 8 hypermutant tumors were excluded. P = 0.891 was determined by Welch’s t-test. [file 40001_2023_1207_MOESM2_ESM.docx]

Figure S2. The average mutation density between HPV-positive and HPV-negative PCa groups when the 8 hypermutant tumors were excluded. *P* = 0.891 was determined by Welch’s t-test.
