# Supplementary figures and images for: Genomic alterations related to HPV infection status in a cohort of Chinese prostate cancer patients
Source: Eur J Med Res. 2023 Jul 17;28:239. doi: 10.1186/s40001-023-01207-2 (PMC10351112; doi:10.1186/s40001-023-01207-2)

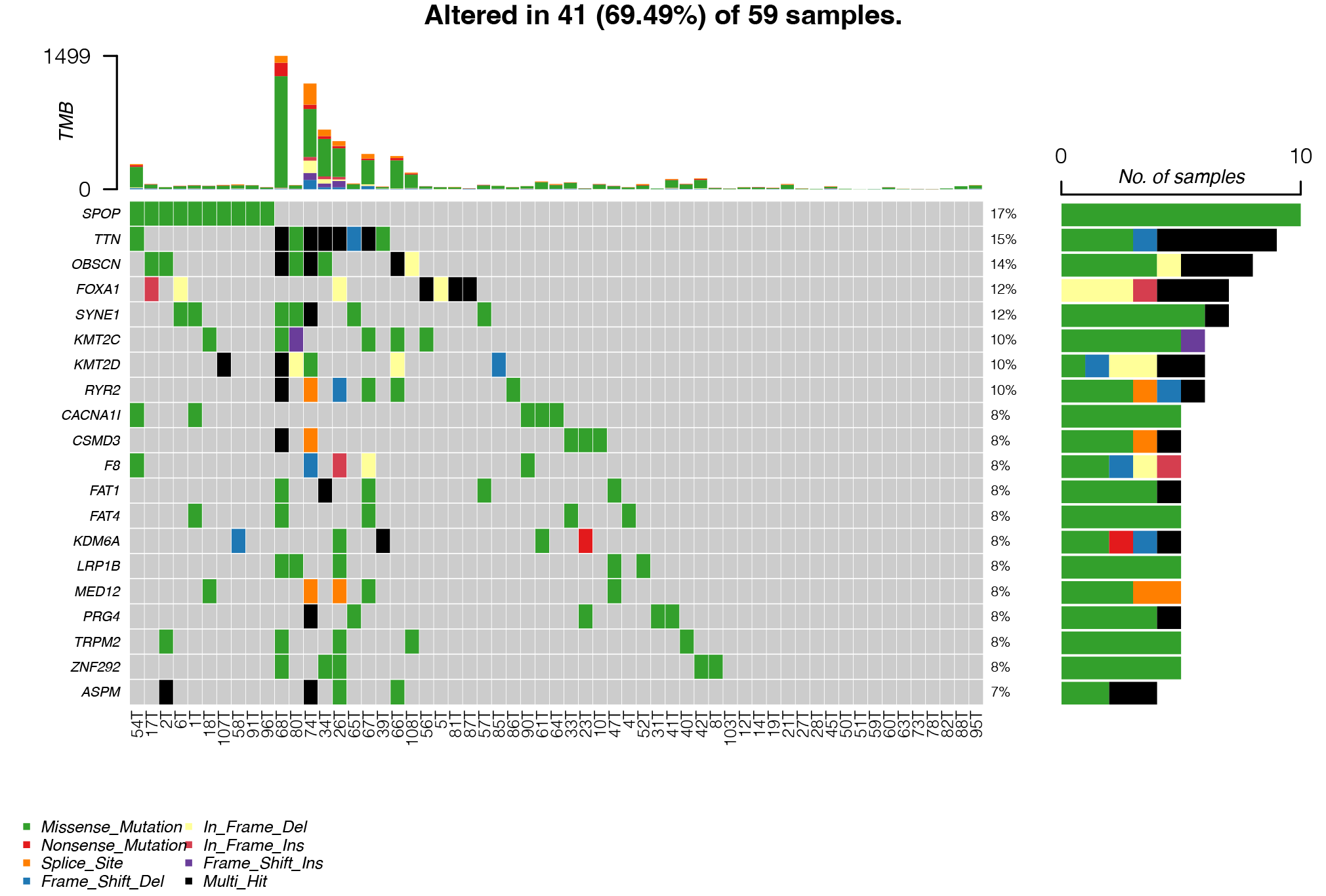


Figure S3. Oncoplot depicting the most recurrent somatic mutations in PCa cohort.

Supplement: Supplementary file 3 — Additional file 3: Figure S3. Oncoplot depicting the most recurrent somatic mutations in PCa cohort. [file 40001_2023_1207_MOESM3_ESM.docx]
